# CT/MRI-based finite element model construction and biomechanical analysis of acetabular quadrilateral fractures

**DOI:** 10.1038/s41598-025-32980-1

**Published:** 2025-12-19

**Authors:** Jieyu Chen, Huawu Liu, Lei Bai, Sergei V. Petrenko, Chaohui Wang, Jianhui Yan, Yonglin Ge

**Affiliations:** 1 School of Basic Medicine, Xiangnan University, Chenzhou, 423000 Hunan China; 2 International Sakharov Environmental Institute of Belarusian State University, Minsk, 220000 Belarus; 3 Department of Orthopaedic Trauma, Chenzhou No.1 People’s Hospital, Chenzhou, 423000 China; 4 The Affiliated Hospital of Xiangnan University, Chenzhou, 423000 Hunan China

**Keywords:** Acetabular quadrilateral plate, Fracture mapping, Finite element analysis, Internal fixation, Anatomy, Diseases, Health care, Medical research

## Abstract

The study aims to construct a representative acetabular quadrilateral fracture model and compare the biomechanical performance of three internal fixation techniques. CT data from 70 fractures were analyzed to create fracture line mappings. A finite element model incorporating ligaments, cartilage, and fracture lines was developed using CT/MRI data from a healthy volunteer. Five loading conditions (standing, supine, lateral lying, sitting, femoroacetabular impingement) were simulated. Four groups were compared: unfixed (Group A), posterior column screw fixation (Group B), quadrilateral buttress screws fixation (Group C), and combined posterior column and quadrilateral buttress screws fixation (Group D). The most frequent fractures were both-column and anterior wall fractures. Fracture line displacement under standing conditions was Group A > Group B > Group C > Group D. In other conditions, the trend was Group A > Group C > Group B > Group D. Peak stress on posterior column screws occurred in the supine position for Group D, while Group B had greater stress in other conditions. Quadrilateral buttress screws showed peak stress in lateral lying for Group D, but Group C had greater stress in other conditions. The highest stress among cortical screws fixed via plates occurred in the posterior screws. Combined posterior column and quadrilateral buttress screws fixation may provide superior mechanical stability and could be considered as a preferred option for clinical fixation of acetabular quadrilateral fractures, pending further clinical validation. Placement of cortical screws near the fracture line is recommended, with additional screws at the sacroiliac joint if necessary. Early postoperative weight-bearing should be approached with caution, based on biomechanical evidence.

## Introduction

 Acetabular fractures account for approximately 0.8% of all fractures, with a higher incidence in females than in males^1^. With the increasing development of transportation and growing aging population, the incidence of acetabular quadrilateral plate fractures has been steadily rising. For unstable quadrilateral plate fractures, the primary clinical treatment involves open reduction and internal fixation. Commonly employed fixation methods include posterior column screws, quadrilateral buttress screws, and a combination of both. Due to the deep anatomical location of the acetabulum and the thin medial wall of the quadrilateral plate, adjacent to critical neurovascular structures, quadrilateral fractures are frequently comminuted, making anatomical reduction challenging. Improper screw placement risks breaching the articular cavity^2,3^. Consequently, selecting an appropriate fixation strategy remains a crucial topic in orthopedic research.

Fracture mapping is a computational approach that overlays multiple fracture lines onto a standardized model, facilitating a visual representation of fracture trajectories and distributions. This technique has been extensively utilized for Pilon fractures, tibial plateau fractures, and proximal humeral fractures^4^. Finite element analysis (FEA) is a numerical method that discretizes a structure into smaller elements to simulate and solve physical problems mathematically. It has become a well-established tool in fracture treatment, implant design validation, and surgical planning^5–8^. Currently, there is no relevant research on using fracture line mapping to plot high-frequency fracture lines of the acetabulum and combining finite element analysis to explore the fixation effects of different internal fixation methods on these fracture lines. Hereby, this study retrospectively analyzed CT data from 70 patients with acetabular fractures to generate a fracture mapping and identify common fracture patterns. A representative quadrilateral fracture model was then constructed based on CT and MRI data from a healthy adult male. Using finite element modeling, we evaluated the biomechanical performance of three common internal fixation techniques under five different loading conditions: standing, supine, lateral lying, sitting, and femoroacetabular impingement. The objective was to provide clinical recommendations for the optimal fixation strategy for quadrilateral plate fractures.

## Materials and methods

### Patient selection and data collection

A retrospective analysis was conducted on 70 cases of acetabular fractures from December 2020 to December 2023 at The Affiliated Hospital of Xiangnan University and The First Affiliated Hospital of Xiangnan University. Among participants, 50 were male, and 20 were female, with an age range of 14 to 86 years (mean age: 47.66 ± 16.09 years). The inclusion criteria comprised pure acetabular fractures and high-resolution CT scans with slice spacing ≤ 2.0 mm and thickness ≤ 2.7 mm. The exclusion criteria included diagnosed acetabular deformities or dysplasia, incomplete closure of acetabular epiphyseal plates, a history of acetabular surgery, and poor-quality CT images with unclear boundaries. This study was approved by the Medical Ethics Committee of The Affiliated Hospital of Xiangnan University (Approval No. K2024-008-01). All procedures were performed in accordance with the Declaration of Helsinki and relevant guidelines and regulations. Written informed consent was obtained from all participants.

### Healthy volunteer CT and MRI data acquisition

A 21-year-old adult male volunteer ( weight: 60 kg, height: 178 cm) with no history of lumbar, pelvic, or hip joint disorders was recruited. CT scanning was performed using a 256-slice spiral CT scanner while the volunteer was supine, capturing 2,993 slices with a thickness of 0.63 mm. MRI scanning was conducted with a Signa HDx 3.0T MRI scanner, covering the pelvis, bilateral proximal femurs, and L_4_/L_5_ vertebrae, producing 50 slices with a thickness of 1.3 mm.

### Development of the lumbar-pelvis-hip joint 3D model

**Fig. 1:**
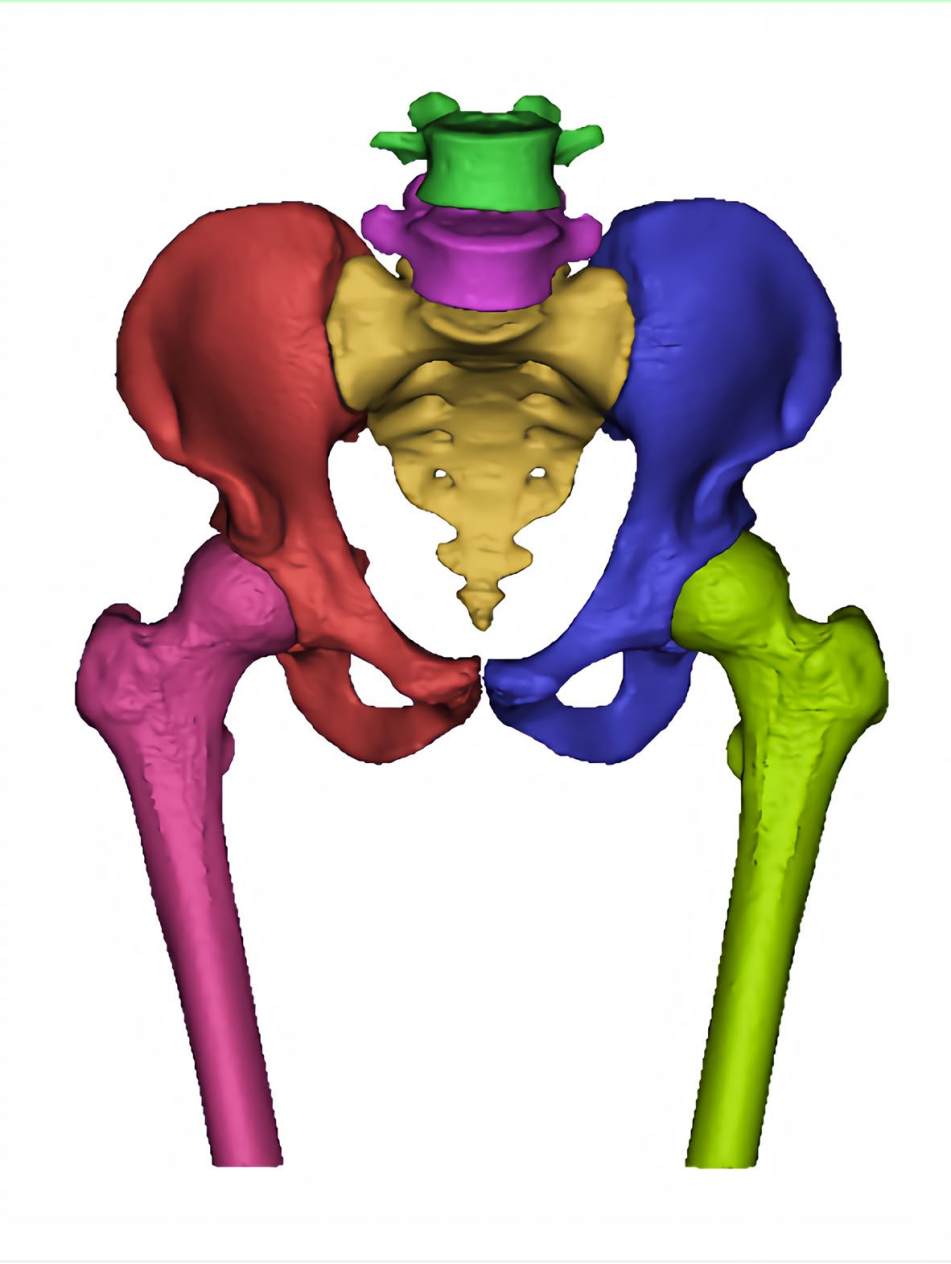
Three-dimensional modeling of lumbar spine-pelvis-hip joint bony structure.

**Fig. 2:**
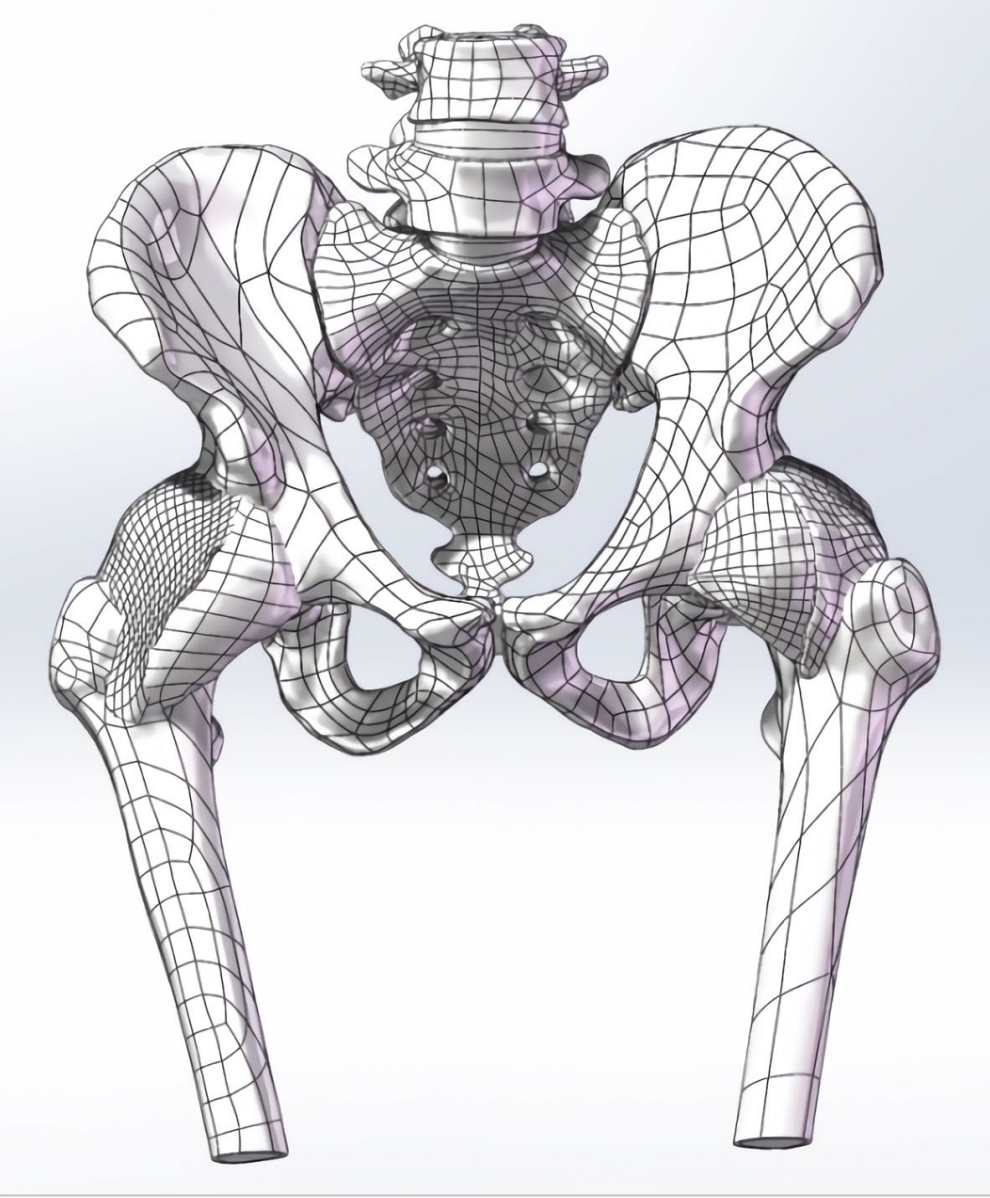
Three-dimensional solid model of lumbar spine-pelvis-hip joint containing soft tissue.

**Fig. 3:**
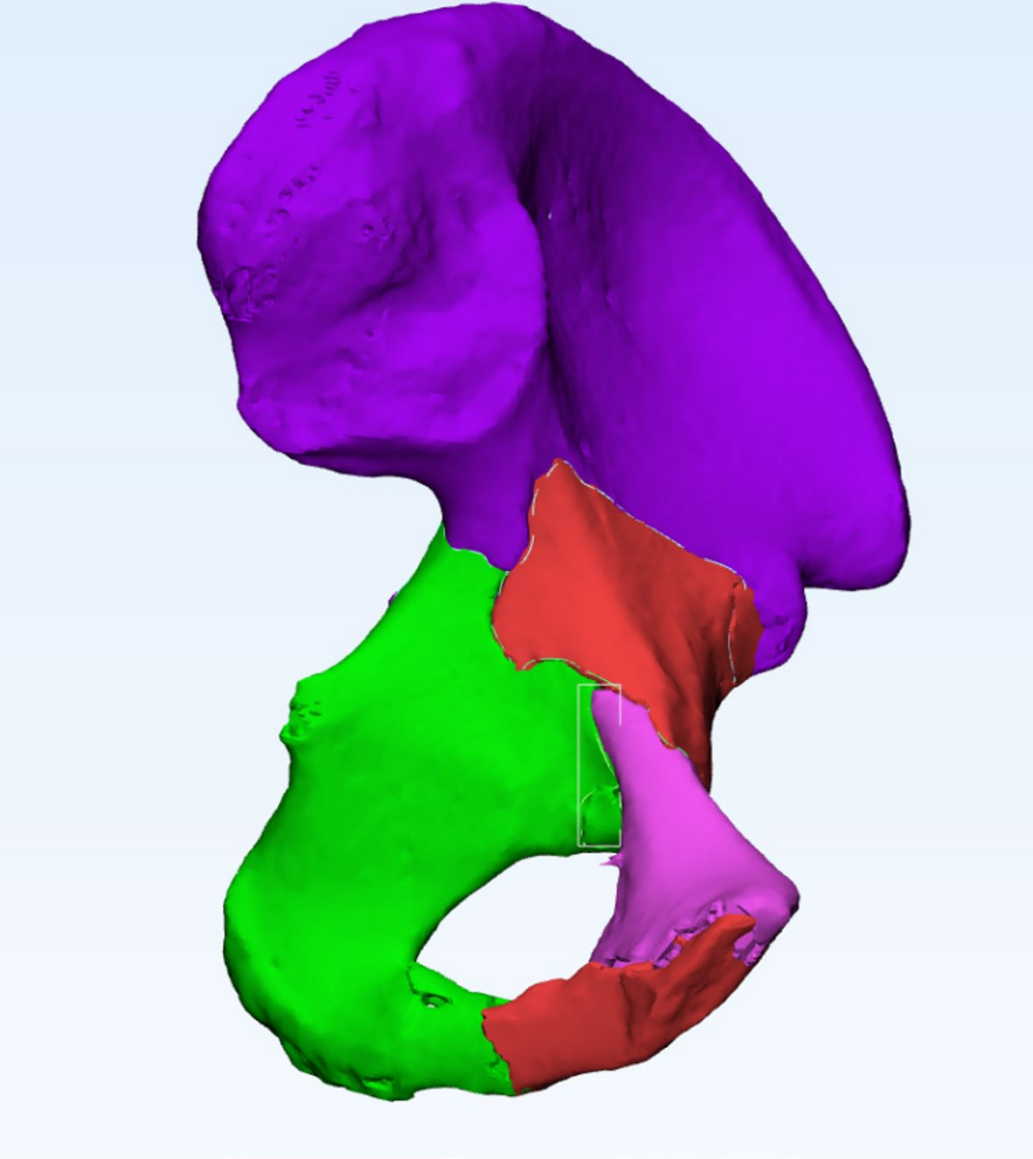
Schematic diagram of bone block repositioning in a patient with acetabular quadrilateral body fracture.

**Fig. 4:**
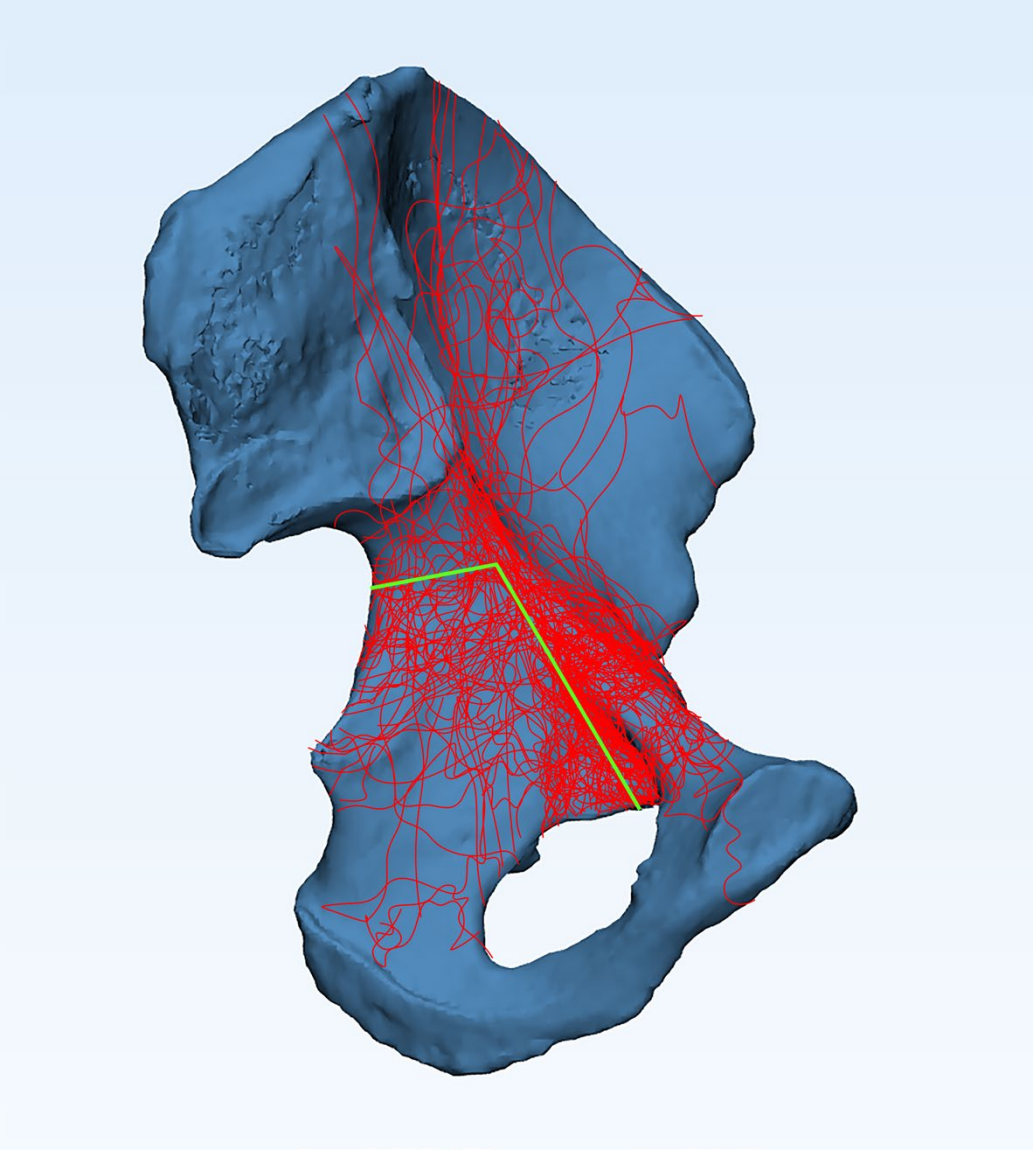
Schematic diagram of the typical fracture line.

Mimics Medical 14.0 (Materialise, Belgium) was employed for the three-dimensional reconstruction of pelvic structures, including the lumbar spine, pelvis, and femoral structures based on CT imaging (Fig. 1). In contrast, the pubofemoral, iliofemoral, and ischiofemoral ligaments were analyzed using MRI (Fig. 2). The software 3-matic was utilized for the repositioning of fractured bone fragments in 70 cases of acetabular fractures (Fig. 3). Additionally, Adobe Photoshop 2018 (Adobe Systems, USA) was used to manually map fracture lines onto the reference model and to design typical fracture lines (Fig. 4), facilitating the visualization of common patterns and the identification of quadrilateral plate-involved fractures.

**Fig. 5:**
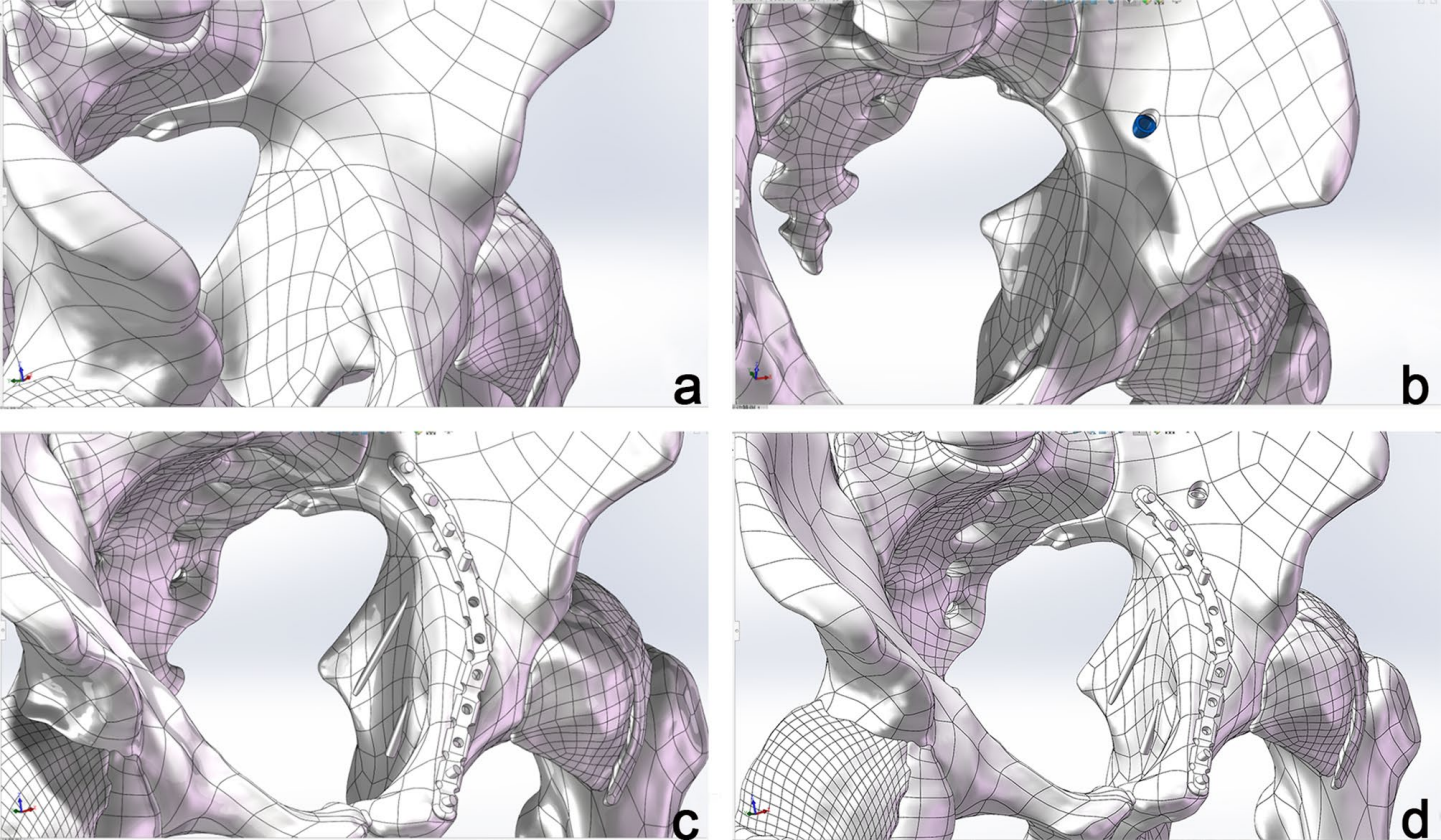
Schematic diagram of Groups A, B, C and D. (**a**) unfixed group; (**b**) a posterior column screw fixation group; (**c**) quadrilateral buttress screws fixation group; (**d**) combined posterior column and quadrilateral buttress screws fixation group.

Smoothing and refinement of the pelvic model were conducted using Geomagic Wrap 2021 (Geomagic, USA). To distinguish between cortical and cancellous bone, the cortical layer was offset 2 mm inward. The design of the cartilage structures and internal fixation devices was completed using SOLIDWORKS 2023 (SolidWorks, USA). For the typical fracture line that we designed, we established four experimental groups to compare the effects of internal fixation (Fig. 5). The groups are as follows: an unfixed group (Group A), a posterior column screw fixation group (Group B), quadrilateral buttress screws fixation group (Group C), and a combined posterior column and quadrilateral buttress screws fixation group (Group D). The details of the fixation are as follows: quadrilateral buttress screws (diameter: 3.5 mm, length: 80 mm); standard cortical screws (diameter: 3.5 mm, length: 40 mm); posterior column hollow screws (diameter: 7.3 mm, length: 100 mm, thickness: 1.15 mm).

### FE model establishment and material property setting

**Fig. 6:**
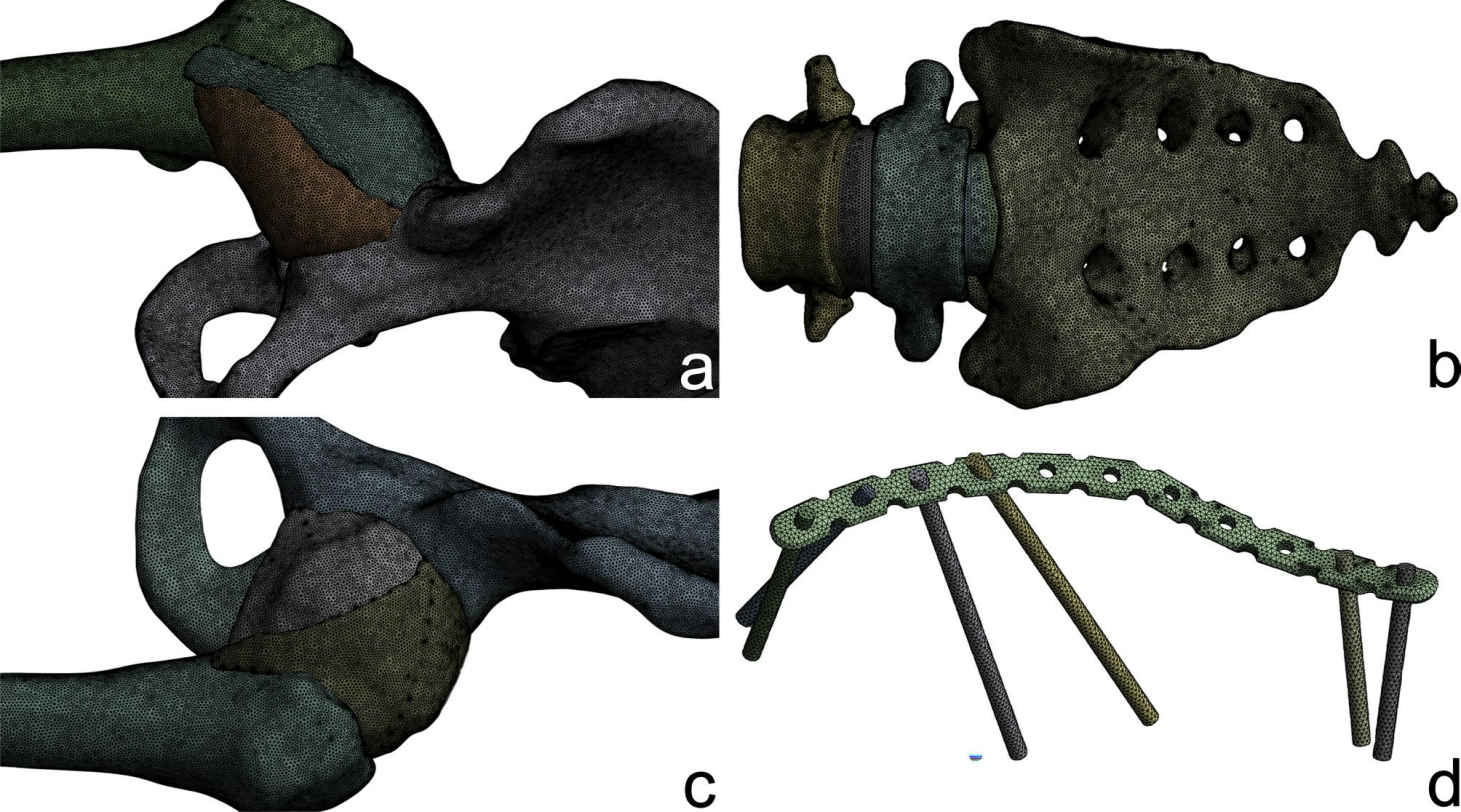
Mesh division diagram. (**a**) Right hip joint; (**b**) sacrum and lumbar spine; (**c**) left hip joint; (**d**) internal fixation.

We utilized ANSYS 17.0 (ANSYS, USA) for finite element analysis and post-processing. The model was imported into ANSYS and characterized as an isotropic linear material based on values from the literature^1,9–13^. Ligament stiffness coefficients were assigned as specified in Table 1. Ligaments were modeled as linear isotropic elastic materials or springs. It is acknowledged that this is a simplification of their tension-only biological behavior, which may introduce artificial stiffening under compressive loads. To verify the mesh independence of the computational results and optimize computational resources, a mesh convergence analysis was conducted. Models with global sizes of 3 mm, 2 mm, and 1 mm were compared, revealing that the stresses in the internal fixation devices and key bony regions such as the acetabulum and sacrum were more sensitive to mesh density. Consequently, a hybrid mesh strategy with local refinement was adopted: the sacrum, lumbar spine, left ilium (cortical and cancellous bone), and internal fixation devices were discretized with 1.0 mm tetrahedral elements, while all other bony structures, cartilage, and ligaments were assigned a 2.0 mm element size (Fig. 6). The final mesh quality varied among the different groups: Group A included 2,257,270 elements and 3,960,161 nodes; Group B comprised 2,285,474 elements and 4,011,568 nodes; Group C contained 2,294,378 elements and 4,028,962 nodes; and Group D consisted of 2,323,101 elements and 4,081,141 nodes.

**Table 1:** Material properties of each part^1,9–13^.

Materials	Young’s modulus(MPa)	Poisson’s ratio(%)	Stiffness coefficient K (*N*/mm)
Cartilage (symphysis pubis)	5	0.45	–
Cartilage (sacroiliac joint)	54	0.4	–
Cartilage (Acetabular cartilage)	12	0.42	–
Cartilage (caput femoris cartilage)	10.5	0.3	–
Titanium steel	114,000	0.3	–
Cortical bone (iliac)	17,000	0.3	–
Cancellous bone (iliac)	132	0.2	–
Cortical bone (sacrum)	6140	0.3	–
Cancellous bone (sacrum)	1400	0.3	–
Cortical bone (femur)	12,000	0.3	–
Cancellous bone (femur)	100	0.3	–
Cortical bone (Lumbar spine)	12,000	0.3	–
Cancellous bone (Lumbar spine)	345	0.2	–
Anuulus fibrosus	55	0.3	–
Nucleus pulposus	1	0.495	–
Endplate	1000	0.4	–
Ischiofemoral ligament	99.5	0.4	–
Pubofemoral ligament	98	0.4	–
Iliofemoral ligament	113.3	0.4	–
Sacrospinous ligament	–	–	1400
Sacrotuberous ligament	–	–	1500
Sacroiliac anterior ligament	–	–	700
Sacroiliac posterior ligament	–	–	1000
Inguinal ligament	–	–	250
Iliolumbar ligament	–	–	1000

**Table 2:** 70 cases of acetabular fractured patients characteristics.

Patient characteristics				*n* = 70
Age				47.66 ± 16.09
Gender				
Male				50 (71%)
Female				20 (29%)
Side of injury				
Left				43 (61%)
Right				27 (39%)
Letournel classification				
Simple fracture				
Anterior wall fracture				17 (24%)
Posterior wall fracture				2 (3%)
Anterior column fracture				4 (6%)
Posterior column fracture				1 (1%)
Transverse fracture				8 (11%)
Complex fracture				
T-shaped fracture				4 (6%)
Posterior column and posterior wall fractue				4 (6%)
Anterior column and anterior wall fractue				2 (3%)
Anterior column and posterior hemi-transverse fracture				6 (9%)
Transverse and posterior wall fractue				5 (7%)
Both-column fracture				17 (24%)
Lesion mechanism				
Low-energy truma				27 (39%)
Traffic accident				36 (51%)
Fall from height				4 (6%)
Injured by weight				2 (3%)
Hurt by somebody				1 (1%)

**Fig. 7:**
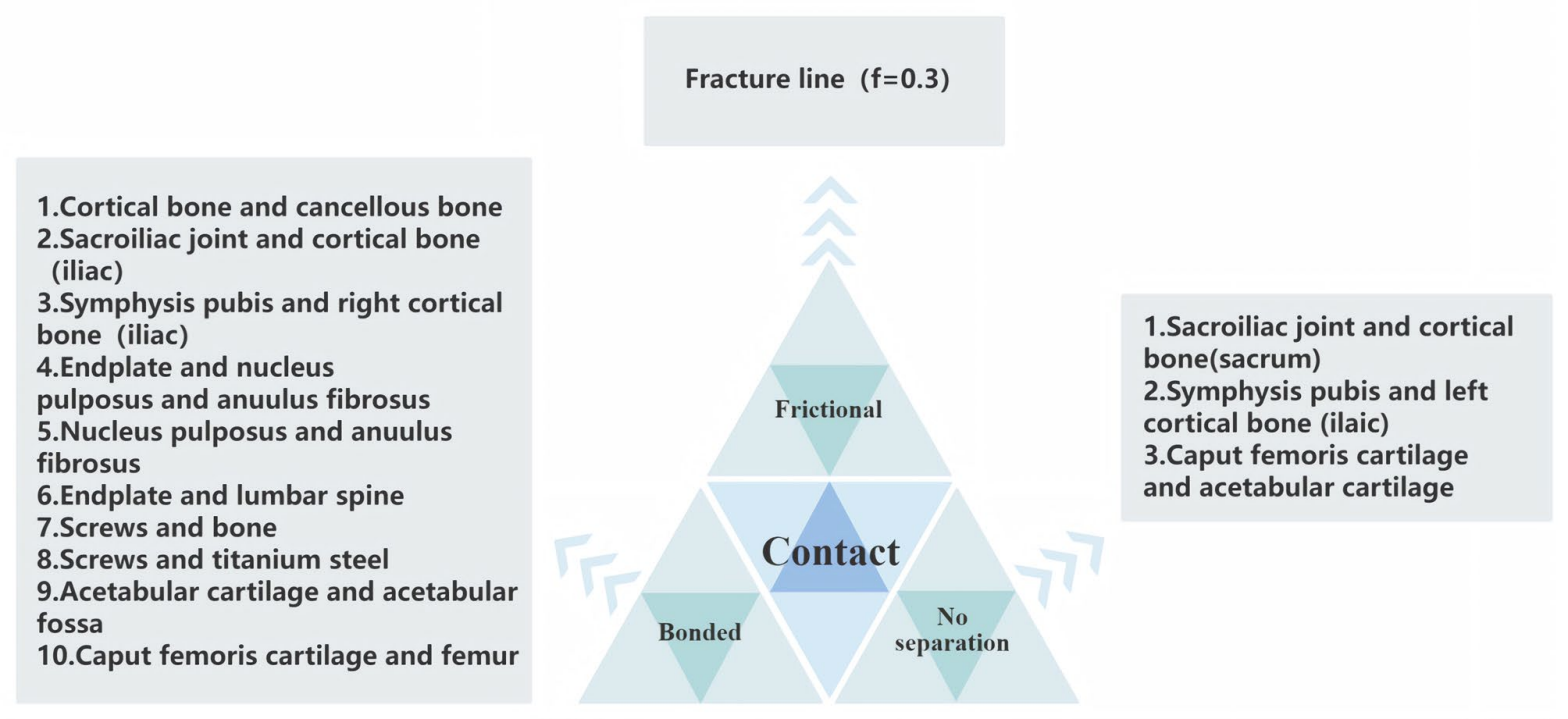
Contact adjustment chart.

**Fig. 8:**
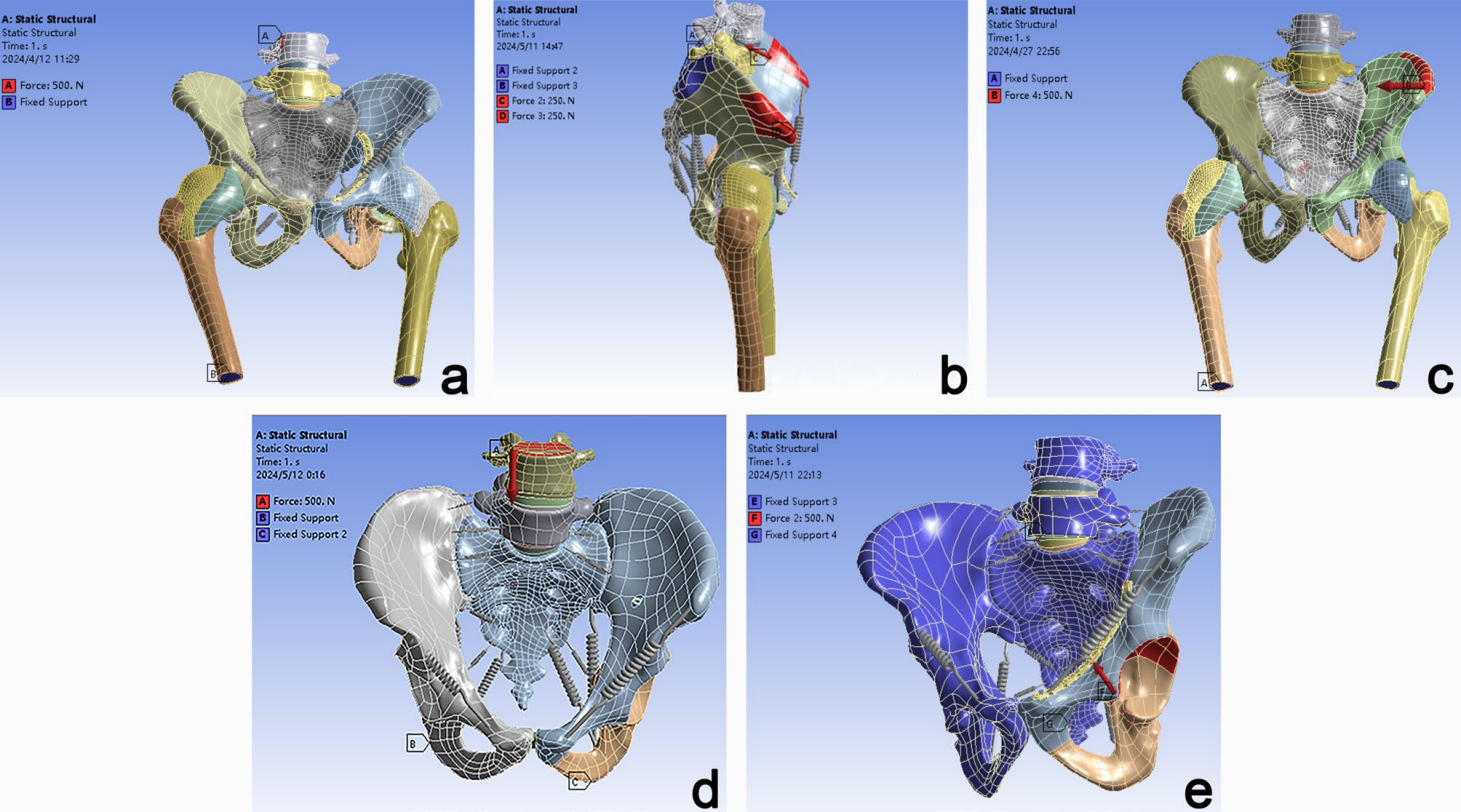
Five groups of working conditions simulation setup diagram. (**a**) Standing; (**b**) Supine; (**c**) Lateral lying; (**d**) Sitting; (**e**) Femoroacetabular impingement.

Contact interactions were defined (Fig. 7), and five physiological loading conditions were simulated (Fig. 8). In the standing condition, both femoral distal ends were constrained, and a vertical force of 500 N was applied at L_4_. For the supine condition, the bilateral iliac crests were constrained, with a force of 250 N applied to the anterior iliac crest. In the lateral lying condition, both femoral distal ends were constrained, and a horizontal force of 500 N was applied to the iliac crest. During the sitting condition, both femurs were constrained, and an axial force of 500 N was applied at L4. Lastly, for femoroacetabular impingement, both femurs were constrained, and a force of 500 N was applied to the acetabular fossa in the direction of the femoral neck.

### Main observation indicators

**Fig. 9:**
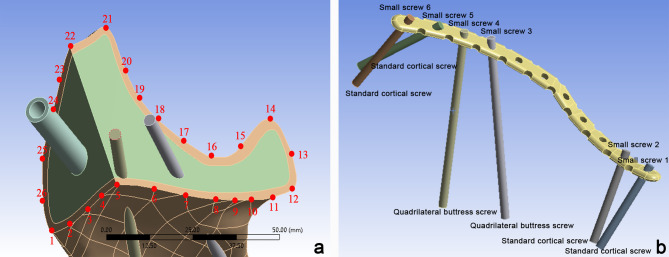
Schematic diagram of point selection and screw naming. (**a**) Schematic of the 26 point selections; (**b**) Schematic of the small screws.

The key observational indicators include the relative displacement along the fracture line and the stress distribution of the fixation system. Specifically, 26 points were selected at the intersections of two fracture lines, with intervals of 5 to 10 mm along the fracture path (Fig. 9a). The relative displacement was determined by calculating the difference between the maximum and minimum displacements. Additionally, the maximum equivalent von Mises stress and its distribution were analyzed for both the internal fixation device and the small screws (1–6) to evaluate their mechanical performance (Fig. 9b).

### Statistical analysis

Fracture displacement data were transformed using square root normalization and analyzed using one-way ANOVA in SPSS 26.0. Data were reported as Mean ± standard deviation (SD), with *P* < 0.05 considered statistically significant.

## Results

### Acetabular fracture classification and distribution

A total of 70 acetabular fracture cases were analyzed, comprising 43 fractures on the left side and 27 on the right. According to the Judet-Letournel classification, the most prevalent fracture types were anterior wall fractures and both-column fractures, each accounting for 24% of the cases (Table 2). Fracture mapping (Fig. 4) revealed that the highest-density fracture zones were located at the arcuate line and the pubic obturator edge, with the eminentia iliopectinea being the most frequently fractured site. This phenomenon may be attributed to the annular structure of the pelvis, which primarily transmits forces from the lumbar spine, rendering the arcuate line particularly susceptible to injury. Furthermore, the structurally weaker iliopubic ramus, which connects the pubic bone to the ilium, is highly vulnerable to fractures resulting from femoral impingement forces. The second highest fracture density is observed between the middle of the greater sciatic notch and the arcuate line, while fractures extending from the sciatic spine to the lower border of the quadrilateral plate are distributed more sparsely. This distribution indicates that the posterior column, particularly its upper region, is more prone to fractures when the femoral head impacts obliquely upward, either due to high-energy trauma or low-energy falls.

### Mesh convergence analysis

**Fig. 10:**
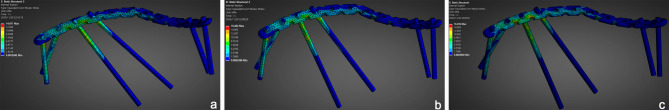
The maximum equivalent von Mises stress diagrams for internal fixtures under three FEA meshing conditions. (**a**) 3 mm; (**b**) 2 mm; (**c**) 1 mm.

To verify the mesh independence of the computational results, a systematic mesh convergence analysis was conducted on key mechanical parameters, with the convergence criterion defined as a variation of less than 5% in critical outputs after successive mesh refinements. The maximum equivalent von Mises stress in the internal fixation was recorded as 14.011 MPa at a mesh size of 3.0 mm, 15.822 MPa at 2.0 mm, and 15.816 MPa at 1.0 mm, with a negligible variation of less than 0.04% between the 2.0 mm and 1.0 mm configurations (Fig. 10). The mean and standard deviation of fracture line relative displacements were 0.003 ± 0.002 mm at 3.0 mm, 0.004 ± 0.003 mm at 2.0 mm, and 0.004 ± 0.003 mm at 1.0 mm, showing consistent values between the 2.0 mm and 1.0 mm mesh configurations. Under a 500 N load, the overall mechanical response of the model remained consistent; although mesh refinement captured more localized deformations, the computed displacements exhibited reasonable and continuous variation without abnormal fluctuations, indicating controllable and predictable global mechanical behavior. These results confirm that the adopted mesh configuration demonstrates adequate convergence.

### Fracture line displacement

**Fig. 11:**
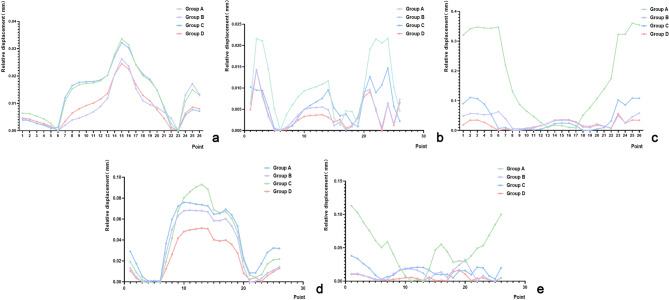
Relative displacement of fracture line paths for five groups of working conditions fracture line plot. (**a**) Standing; (**b**) supine; (**c**) lateral lying; (**d**) sitting; (**e**) femoroacetabular impingement.

The relative displacement of the fracture line under standing conditions exhibited a clear trend: Group A > Group B > Group C > Group D (*F* = 216.744, *P* < 0.01) (Fig. 11a). In the remaining four conditions: supine (*F* = 220.641, *P* < 0.01), lateral lying (*F* = 196.204, *P* < 0.01), sitting (*F* = 205.071, *P* < 0.01), and femoroacetabular impingement (*F* = 213.206, *P* < 0.01), the displacement pattern was Group A > Group C > Group B > Group D (Fig. 11b–e).

### Maximum equivalent von mises stress on fixation devices

#### Posterior column screws

**Fig. 12:**
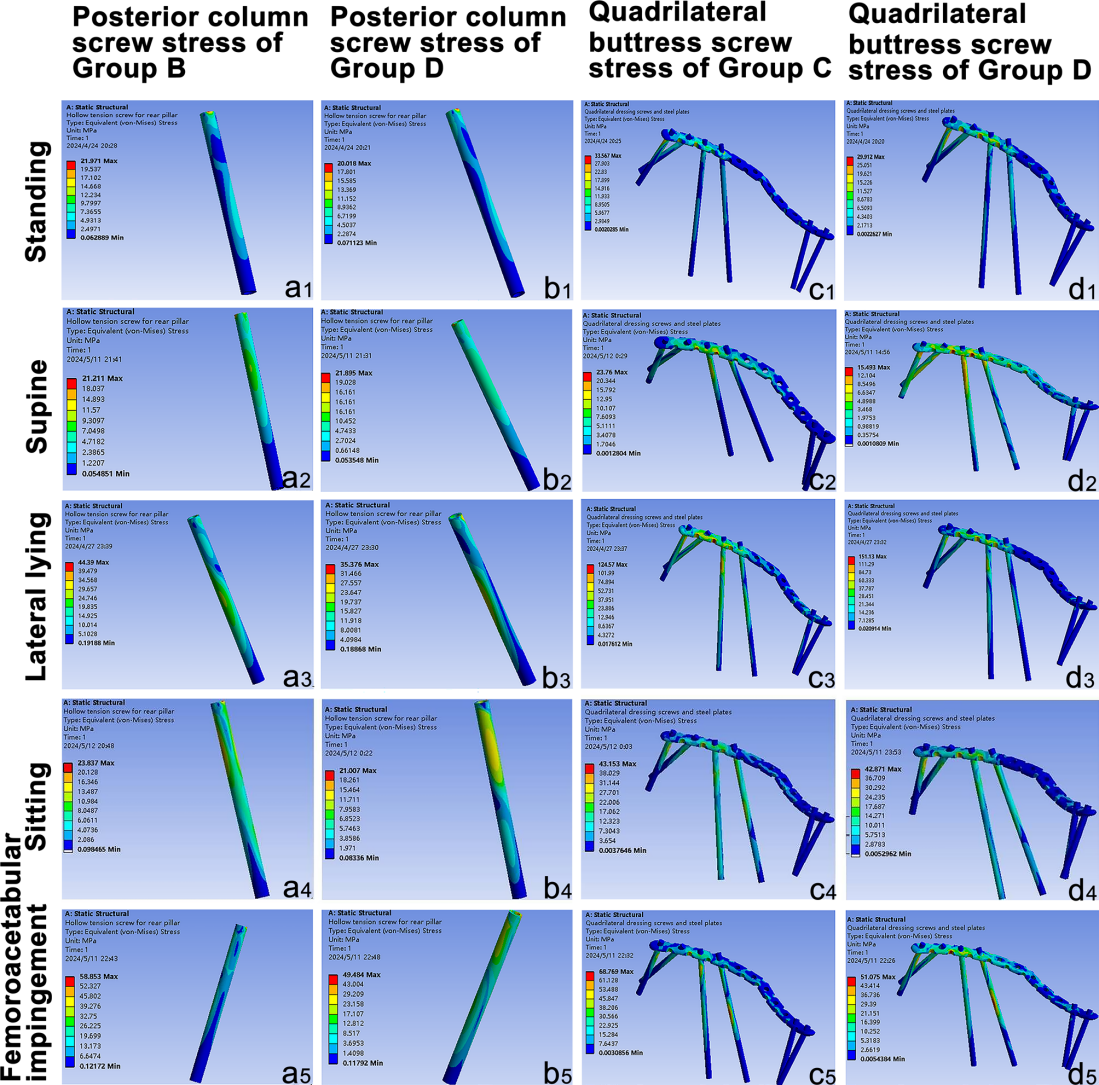
The maximum equivalent von Mises stress diagrams for internal fixtures under five operating conditions. (**a**_1–5_), posterior column screw stress of Group B; (**b**_1–5_), posterior column screw stress of Group D; (**c**_1–5_), quadrilateral buttress screws and plate stress of Group C; quadrilateral buttress screws and plate stress of Group D; (**a**_1_–**d**_1_), standing; (**a**_2_–**d**_2_), Supine; (**a**_3_–**d**_3_), lateral lying; (**a**_4_–**d**_4_), sitting; (**a**_5_–**d**_5_), femoroacetabular impingement.

Under supine loading, Group D exhibited higher stress levels than Group B (Fig. 12-a_2_, b_2_). However, in the remaining conditions, Group B demonstrated greater stress than Group D (Fig. 12-a_1_, a_3 − 5_, b_1_, b_3 − 5_).

#### Quadrilateral buttress screws

In the lateral lying position, Group D experienced higher stress than Group C (Fig. 12-c_3_, d_3_), while Group C exhibited greater stress in the other four conditions (Fig. 12, c_1 − 2_, c_4 − 5_, d_1 − 2_, d_4 − 5_).

#### Cortical screws fixed via plates

Across all conditions, posterior column screws endured greater stress than anterior screws, with the highest stress levels recorded in screws 5 and 6 (Table 3).

**Table 3:** The maximum Von-mises stress of common screws (MPa).

Group	Screw	Posture				
		Standing	Supine	Lateral lying	Sitting	Femoroacetabular impingement
Group C	C_1_	13.294	2.3086	22.185	2.507	11.159
	C_2_	12.746	1.8624	12.375	8.5024	11.566
	C_5_	14.905	9.5625	54.582	20.632	29.78
	C_6_	22.195	14.29	105.890	35.52	48.487
Group D	D_1_	10.530	2.0963	31.878	3.3426	11.439
	D_2_	8.929	1.6074	20.287	5.2086	11.943
	D_5_	13.971	8.2172	36.182	19.127	23.568
	D_6_	25.521	12.509	76.546	36.649	49.087

## Discussion

The acetabular quadrilateral plate, the “third column” alongside the anterior and posterior columns, is essential for maintaining normal weight-bearing function^14^. According to the Clinical Diagnosis and Treatment Guidelines for Acetabular Quadrilateral Plate Fractures (2023)^3^, fixation strategies involve either partially or completely blocking the quadrilateral plate after stabilizing the anterior and posterior columns using a “frame” structure. Various internal fixation devices have been designed and analyzed through finite element methods, including periacetabular channel screws^15^, dynamic anterior plating systems^16^, customized 3D-printed blocking plates^17^, and specialized quadrilateral plate-integrated anatomical plates^9,18^. However, no standardized treatment protocol currently exists, and most approaches depend on clinical experience. This study utilized CT and MRI data from a healthy volunteer to construct a three-dimensional finite element model, comparing the mechanical characteristics of an unfixed group with three fixation methods under five loading conditions.

Fracture line mapping technology has been extensively applied to analyze fracture distributions, aiding in classification refinement, surgical planning, internal fixation design, and finite element validation, while providing a theoretical foundation for surgical optimization^4,19,20^. Although previous studies have characterized acetabular fractures using this technique^19^, dedicated investigations into quadrilateral plate fractures remain lacking. This study analyzed CT data from 70 cases of acetabular quadrilateral plate fractures to construct a fracture line map, classifying cases using the Judet-Letournel system to identify high-incidence patterns and distribution trends. The findings indicate that both-column and anterior wall fractures are the most prevalent. A study of 3,346 acetabular fractures reported a 21.2% incidence of both-column fractures^21^, which aligns with this study’s findings (17/70, 24%). Traffic accidents emerged as the predominant injury mechanism, likely due to the characteristic pathophysiology of both-column fractures, where forceful anterior and medial femoral head impact against the acetabular medial wall results in complete dissociation from the axial skeleton. While anterior wall fractures are less common, they are associated with poorer prognostic outcomes^22^ and their higher incidence in this study may be attributable to limitations in sample size.

Finite element analysis demonstrated that the unfixed group (Group A) exhibited the greatest displacement of the fracture line among the five conditions, thereby validating the reliability of the fixation models, consistent with the findings of Fan et al.^23–25^. In contrast, Group D exhibited the minimum displacement, indicating that the combined fixation using posterior column screw and quadrilateral buttress screws provides superior stability. In the standing position, quadrilateral buttress screws fixation (Group B) outperformed posterior column screw fixation (Group C). However, under other conditions, the posterior column screw provided enhanced stability. These findings suggest that posterior column screws effectively mitigate displacement under various loads and should be prioritized for internal fixation, preferably in combination with quadrilateral buttress screws. Furthermore, early postoperative standing and weight-bearing should be minimized, especially for patients fixed with quadrilateral buttress screws, in accordance with Oldhoff et al.^26^, although this approach differs from the recommendations of Berk et al.^27^.

The stability of internal fixation devices is closely related to their maximum equivalent Von Mises stress, with peak stress serving as a key indicator of fixation reliability. Finite element analysis revealed that, except in the supine condition, the posterior column screw in Group B exhibited higher peak stress than that in Group D across four loading conditions, suggesting improved stress distribution and enhanced fixation stability. However, in the supine condition, Group D demonstrated higher peak stress, indicating that quadrilateral buttress screws provide limited resistance to forces at the anterior superior iliac spine level. For quadrilateral buttress screws, peak stress was higher in Group D than in Group C under the lateral lying condition, while in other conditions, Group C exhibited higher stress, suggesting that both fixation methods contribute to stability in this position. Mechanically, plate fixation prevents displacement but lacks angular cross-fixation, whereas posterior column screws provide axial compression and resist shear and torsional forces, although they may not fully prevent fragment displacement when used alone. Combined fixation effectively counteracts multidirectional fragment displacement and enhances overall stability. Based on these findings, the combined use of posterior column screws and quadrilateral buttress screws is recommended as the preferred internal fixation strategy.

The maximum equivalent von Mises stress of small screws represents their fixation strength, with higher peak stress indicating better stability when screw length, diameter, and material remain constant. Across all five loading conditions, cortical screws 5 and 6 consistently exhibited higher peak stress than screws 1 and 2, suggesting that their fixation effect was significantly superior. This may be attributed to the designed fracture line in this study, which primarily involved the acetabular quadrilateral plate and was more concentrated along the posterior column. In light of these findings, it is recommended that clinicians position acetabular peripheral screws closer to the fracture line when applying fixation with a plate. Additionally, depending on intraoperative conditions, increasing the number of screws near the sacroiliac joint may be considered to enhance overall fixation stability.

This study developed a relatively comprehensive finite element model of the lumbar-pelvis-hip joint complex through systematic integration of CT and MRI imaging data. The model reconstruction maintained anatomical continuity by incorporating major solid ligaments and cartilaginous structures, which demonstrated reasonable agreement with actual anatomical features. Based on the analysis of 70 clinical CT cases, a representative fracture line pattern for the acetabular quadrilateral plate was established, thereby improving the clinical relevance of the simulation results. The study evaluated five key rehabilitation postures including standing, sitting, supine, lateral lying, and femoral head impaction, enabling systematic assessment of biomechanical responses under various physiological loading conditions. This integrated methodology provides valuable multi-modal mechanical insights to support clinical rehabilitation strategies, effectively balancing anatomical accuracy with practical clinical applicability.

Several methodological considerations should be noted when interpreting our findings. First, both soft tissues and bony structures were modeled as linear isotropic elastic materials to ensure computational tractability. While this established simplification offers computational efficiency, it inherently does not capture the anisotropic nature of bone or the nonlinear and tension-only properties of biological tissues. Second, a deliberate hybrid modeling strategy was adopted for the ligaments. Specifically, certain ligaments critical for global joint stability were modeled as 3D volumetric solids to preserve their anatomical geometry, while others were represented as spring elements for simplification. It is acknowledged that this pragmatic approach, despite its rationale, may introduce variability and influence the mechanical predictions. Third, potential geometrical inaccuracies could arise from the manual mapping of fracture lines. Finally, the model did not incorporate the effects of surrounding muscles and synovial fluid, thus simplifying the dynamic simulation. Despite these limitations, the current framework provides a valuable and computationally viable method for initial biomechanical investigation. Future work will prioritize the use of computer-generated fracture heatmaps and the implementation of more sophisticated nonlinear material models to enhance biomechanical fidelity.

## Conclusion

To sum up, this biomechanical study suggests that posterior column screw combined with quadrilateral buttress screws fixation may provide superior mechanical stability and could be considered as a preferred option for the clinical fixation of acetabular quadrilateral fractures, pending further clinical validation. Moreover, it is recommended to place cortical bone screws applied through the plate close to the fracture line, and the number of screws may be increased on the side of the sacroiliac joint according to the actual situation. Additionally, based on the current biomechanical findings, early postoperative weight-bearing is not advised.

## Data Availability

The datasets used and analysed during the current study are available from the corresponding author on reasonable request.
